# iADRs: towards online adverse drug reaction analysis

**DOI:** 10.1186/2193-1801-1-72

**Published:** 2012-12-20

**Authors:** Wen-Yang Lin, He-Yi Li, Jhih-Wei Du, Wen-Yu Feng, Chiao-Feng Lo, Von-Wun Soo

**Affiliations:** 1 Department of Computer Science and Information Engineering, National University of Kaohsiung, Kaohsiung, Taiwan; 2 Department of Computer Science and Information Engineering, National Tsing Hua University, Hsinchu, Taiwan

**Keywords:** Adverse drug reaction, Associative classification, Contingency cube, Data mining, Data warehouse, Pharmacovigilance

## Abstract

Adverse Drug Reaction (ADR) is one of the most important issues in the assessment of drug safety. In fact, many adverse drug reactions are not discovered during limited pre-marketing clinical trials; instead, they are only observed after long term post-marketing surveillance of drug usage. In light of this, the detection of adverse drug reactions, as early as possible, is an important topic of research for the pharmaceutical industry. Recently, large numbers of adverse events and the development of data mining technology have motivated the development of statistical and data mining methods for the detection of ADRs. These stand-alone methods, with no integration into knowledge discovery systems, are tedious and inconvenient for users and the processes for exploration are time-consuming. This paper proposes an interactive system platform for the detection of ADRs. By integrating an ADR data warehouse and innovative data mining techniques, the proposed system not only supports OLAP style multidimensional analysis of ADRs, but also allows the interactive discovery of associations between drugs and symptoms, called a drug-ADR association rule, which can be further developed using other factors of interest to the user, such as demographic information. The experiments indicate that interesting and valuable drug-ADR association rules can be efficiently mined.

## Introduction

The WHO definition of an adverse drug reaction (ADR) or adverse drug event (ADE) is an uncomfortable, noxious, unexpected, or potentially harmful reaction resulting from the use of a prescribed medication (WHO 1972). Notably, it refers to a reaction arising from normal doses of drugs for disease prevention, diagnosis, treatment, or for the modification of physiological functions, but it excludes drug withdrawal symptoms, including drug abuse, poisoning or drug overdose. Adverse drug reactions waste many social resources and cause different degrees of psychological or physiological suffering to patients and their relatives. They also limit the efficacy of treatment and waste medical resources, thereby increasing medical costs and decreasing the quality of medical care. Many adverse drug reactions are not discovered through limited pre-marketing clinical trials; instead, they are only seen in long term, post-marketing surveillance of drug usage. In light of this, most developed countries have established spontaneous reporting systems (SRSs) that collect data for suspected adverse drug events for further analysis, e.g., the Adverse Event Reporting System (AERS) of the US Food and Drug Administration (FDA), the Canadian Vigilance Adverse Reaction reporting system (MedEffect Canada), the Australian Adverse Drug Reaction Reporting System. The detection of adverse drug reactions, as early as possible, from these SRSs forms an important research field for the pharmaceutical industry (WHO 2002).

Many studies have detected possible adverse drug reactions or have analyzed the factors relevant to adverse drug reactions. These studies can generally be divided into two categories. The first category uses statistical or data-mining methods to identify the signals of adverse drug reactions (Beta et al. 1998; Dumouchel 1999; Evans et al. 2001; Huang et al. 2007; Jin et al. 2007; Orre et al. 2000; Szarfman et al. 2002). These stand-alone methods, without any integration with knowledge discovery systems, are tedious and inconvenient for users to use to identify possible adverse drug reactions. The other category uses adverse drug reactions exploration systems (Fram et al. 2003). Although these systems can be used to identify possible adverse drug reactions, it takes a long time to obtain the results of each exploration run, so any examination from different viewpoints is very time-consuming.

This study develops a platform to analyze adverse drug reactions, which combines data warehousing and data mining technologies, through which users can observe and analyze drug-ADR signals from different viewpoints. Specifically, a contingency-cube-based method and an associative-classification-based method are proposed to facilitate the interactive detection of suspected drug-ADR and multidrug-ADR signals, respectively. The experimental results show that the cube-based approach significantly outperforms an associative-classification-based approach and the interactive exploitation of suspected association of drugs and symptoms from a data warehouse is more efficient.

The remainder of this paper is organized as follows. In Section 2, related work and terminology is described. An overview of the proposed iADRs system platform is presented in Section 3. The proposed contingency-cube-based method and an associative-classification-based method are described in Sections 4 and 5, respectively. Section 6 presents the experiments and describes the current interface and functionalities of the proposed iADRs system. Conclusions and recommendations for future study are presented in Section 7.

### Related work

Recently, the accumulation of large volumes of data related to adverse events and the development of data mining technology have spawned the use of statistical or data mining methods for the detection of ADRs. These methods can be divided into two categories: the measures of disproportionality and the Bayesian methods.

The measures of disproportionality are commonly used techniques for the identification of ADRs. Although different measures for calculating disproportionality are not concordant, they all use a 2×2 contingency table as shown in Table 1. The most common measures include the Proportional Reporting Ratio (PRR) used by the UK Yellow Card database (Evans et al. 2001), the Reporting Odds Ratio (ROR) used by the Netherlands Pharmacovigilance Foundation (Egberts et al. 2002) and the MHRA, an integrated measure used by the UK Medicines and Healthcare products Regulatory Agency (MHRA) (Evans et al. 2001). The MHRA combines the PRR, the numbers reported and a chi-squared test. The definitions of these measures are shown in Table 2.

**Table 1:** **The 2×2 contingency table used for the identification of ADRs**

	Suspected ADR	All other ADRs	Total
Suspected drug	*a*	*b*	*a + b*
All other drugs	*c*	*d*	*c + d*
Total	*a + c*	*b + d*	*a + b + c + d*

**Table 2:** **The measures of proportionality used for the identification of ADRs**

Measure	Formula	Definition
PRR		PRR − 1.96*δ* > 1
ROR		ROR − 1.96*δ* > 1
MHRA	PRR, *a*, χ^2^	PRR ≥ 2, *a* ≥ 3, χ^2^ ≥ 4

*δ: standard error, χ^2^: Yates’ chi-squared test statistic ( [Yates 1934]).

The best-known Bayesian-based method is the Bayesian Confidence Propagation Neural Network (BCPNN) used by the World Health Organization (WHO) (Beta et al. 1998; Orre et al. 2000). This approach uses Bayesian statistics in a neural network architecture and calculates an information component (IC) for each drug–ADR combination. The IC value measures the strength of the association between two variables, a drug *x* and an ADR *y*, which is defined by the following formula:

where *p*(*x*) is the probability of drug *x* in all reports, *p*(*y*) is the probability of ADR *y* in all reports, and *p*(*x*, *y*) is the probability of drug *x* and ADR *y* together in all reports. If the IC value of a drug-ADR pair is higher than a given threshold, the drug is regarded to have a significant association with the ADR.

The US Food and Drug Administration (FDA) uses an algorithm called Empirical Bayes Gamma-Poisson Shrinker (EBGPS) to detect those ADRs that have the frequency of reporting higher than the expected value (Dumouchel 1999). This algorithm also uses a Bayesian statistical formula to calculate the observed reporting value and the expected reporting value for each drug-ADR pair. The observed ratio of reporting value to expected reporting value represents the strength of the signal of the drug-ADR pair. A drug-ADR pair with an observed ratio higher than the threshold is more significant and worthy of further investigation.

An algorithm that adopts the temporal association rule technology, called MUTARC, was proposed by Jin et al. (Jin et al. 2007; Jin et al. 2010). Using a database provided by the Queensland Department of Health, called the Queensland Linked Data Set, an Unexpected Temporal Association Rule (UTAR) was defined to identify ADRs. In other words, if an unexpected event happens within a certain period after treatment, then this event may be an ADR. So, the algorithm must prune the expected events, the patient’s illnesses, before a process of exploration, and use support and leverage to filter and order the association rules. The support for an association rule refers to the percentage of the total reports for which the pattern is true. The leverage of an association rule refers to the measure of dependency between the antecedent (factor) *X* and the consequences (symptom) *Y* of a rule. An association rule with leverage greater than zero signifies that *X* has a positive association with *Y*, a leverage of less than zero signifies that *X* is negatively associated with *Y* and that a leverage equal to zero signifies that *X* is independent of *Y* (Piatetsky-Shapiro 1991). The formula for leverage is defined as follows:

Huang et al. ( 2007) proposed a statistical method that uses the chi-square test and conditional probability. The study sought to determine any drug-drug interaction for the ADRs. Firstly, a chi-square test is used to calculate the dependency of all drug and symptom pairs. The chi-square test, however, can only show the relative strength of association, but cannot distinguish whether the ADR is caused by drug-drug interaction or a single drug. The use of a conditional probability resolves this problem.

Another study by Fram et al. ( 2003) proposed an ADR exploration system. The EBGPS was used to construct a platform that is used to identify the factors of ADRs. This platform allows users to individually determine the factors of ADRs that are of interest.

### The proposed system framework

#### Design strategy

This proposed system establishes an interactive platform for the end user to allow the analysis and detection of suspicious ADR signals. It is well known that ADR signal detection is time consuming - at least of the same complexity as typical data mining tasks, such as association rule mining and classification analysis. In order to reduce the computation time, a general concept commonly used in the context of query processing —pre-computation – is used. This executes the partial or total computation involved in the process of answering the query, in advance.

#### System overview

**Figure 1:**
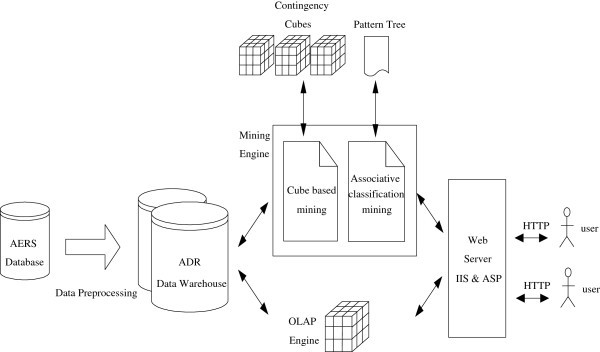
**System architecture of the proposed iADRs.**

As shown in Figure 1, the proposed system, iADRs, relies on a data warehouse repository, to support OLAP analysis, and a mining engine. The data source used is the Adverse Event Reporting System (AERS) database of the US Food and Drug Administration (FDA) ( 2012); the addition of other public data sources such as MedEffect Canada is ongoing. AERS is used to support post-marketing safety surveillance for all approved drugs and therapeutic biochemical products, in order to effectively prevent the occurrence of ADRs. This database, containing more than 3 million clinical reports, was created in 1968 and is released by FDA each quarter. The front-end of the proposed iADRs system contains two main analysis tools for the detection and analysis of ADRs, the OLAP engine and drug-ADR association mining. The OLAP engine allows users to perform multi-dimensional explorations of multidimensional data in a data warehouse (Chaudhuri & Dayal 1997). The association mining engine is used to discover high risk pattern-symptom association rules using the proposed mining algorithms, a contingency cube-based approach for drug reactions and an associative-classification based approach for drug-drug reactions. More complex drug reactions analysis algorithms will be developed in the near future. This system is portable and convenient, because it conforms to a Web platform. Users do not have to install any additional software.

#### ADR warehouse schema

**Figure 2:**
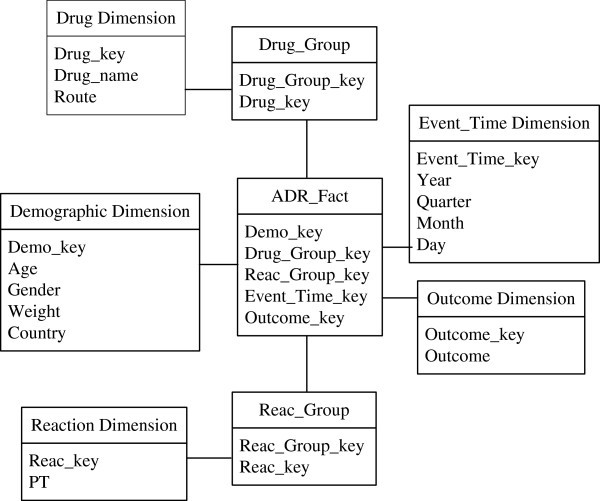
**The star schema of an ADR warehouse.**

Data generated in the clinical medical field are very different to data generated in practice. The data model of a clinical data warehouse is much more complicated than that for business applications (Pedersen & Jensen 1998). For example, the relationship between the fact table and the dimension tables in a multi-dimensional model is one-to-many, while the relationship is many-to-many in clinical data warehouses, i.e., a patient’s anamnesis not only has more than one drug record, but also has more than one symptom record. Since the relationship between drug and symptom is many-to-many, it cannot be effectively modeled using traditional multi-dimensional models, such as star schema. Following the approach recommended by Kimball et al. ( 1998), a medial dimension table, namely a *bridge table*, is used to describe the many-to-many relationship. The schema of the ADR data warehouse, consisting of one fact table, five dimension tables and two bridge tables, is shown in Figure 2.

The fact table, ADR_Fact, comprises five foreign keys; Demo_key, Drug_Group_key, Reac_Group_key, Event_Time_key and Outcome_key, which connect with the Demographic dimension table’s Drug_Group bridge table, Reac_Group bridge table, Event_Time dimension table and Outcome dimension table, respectively. The numerical measure of ADR_fact is count (number of records), which can be omitted, because the number of each record is only one.

The five dimension tables of the ADR data warehouse are Demographic, Event_Time, Outcome, Drug and Reaction, each of which is described as follows.

1. Demographic: Patient demographic and administrative information; a single record for each event report.
2. Event_Time: The date of occurrence of adverse drug reactions. The attributes in this table are related by a total order and form a concept hierarchy, such as “Day < Month < Quarter < Year”.
3. Outcome: The information about the outcome, after therapy.
4. Drug: The information about the patient’s medication record.
5. Reaction: The information about the patient’s symptoms, before therapy. Attribute PT (Preferred Term) refers to adverse reactions, coded using the MedDRA (Medical Dictionary for Regulatory Activities).

The two bridge tables, Drug_Group and Reac_Group, represent the drug records and symptom records of the anamneses, respectively. Drug_Group_key and Reac_Group_key are the identifiers of the drug group and the symptoms group, indicating a patient who has taken many drugs and who has suffered many symptoms.

### Cube-based method for the detection of ADR signals

Since a spontaneous reporting database usually contains a very large amount of data, the detection and analysis of ADRs is a challenging task. Inspired by the success of OLAP operations, which responds to user queries immediately, and the techniques for data cube processing that are well supported by contemporary data warehousing systems, this study uses the OLAP cube technique for its ADRs mining methods. In light of all of the contemporary measures of disproportionality derived from the contingency table (see Tables 1 and 2), the concept of contingency cubes is proposed to refer to the set of pre-stored data cubes for the detection of ADR.

#### Contingency cube

As presented in Section 3.1, the measured values of proportionality for ADR signaling rules can be calculated using the 2×2 contingency table. Further, when a drug-ADR rule is constrained, its measure can be calculated using the corresponding constrained (predicate) contingency table. For example, consider the following drug-ADR rule:

The corresponding contingency table is shown in Table 3.

**Table 3:** **The 2×2 contingency table to identify ACCUTANE-HAMARTOMA constrained by*Age*= 20~60,*Gender*= Male**

***Age*** = 20~60, ***Gender*** = Male	HAMARTOMA	All other ADRs
ACCUTANE	*a*	*b*
All other drugs	*c*	*d*

A contingency cube defined on the ADR star is an *n*-dimensional cube *C*[*A*_1_, *A*_2_, …, *A*_*n*_], where *A*_1_, *A*_2_, …, *A*_*n*_ denote dimensions (attributes) and *A*_*n*−1_ = *Drug*, *A*_*n*_ = *PT*. Except for *A*_*n*−1_ and *A*_*n*_, the value set associated with each dimension, *A*_*i*_, is *domain*(*A*_*i*_) ∪ {*}, where *domain*(*A*_*i*_) denotes the set of distinct values of *A*_*i*_ in the ADR star and “*” denotes “any” or “don’t care”. Note that the value sets associated with *drug* and *PT* do not include “*”, because the defined contingency table is always associated with a specific drug and symptom. Each cell *C*[*a*_1_, *a*_2_, …, *a*_*n*_] in the cube store conceptualizes the corresponding contingency table of *Drug* = *a*_*n*−1_ and *PT* = *a*_*n*_ with constraint *A*_1_ = *a*_1_, *A*_2_ = *a*_2_, …, *A*_*n*−2_ = *a*_*n*−2_.

**Figure 3:**
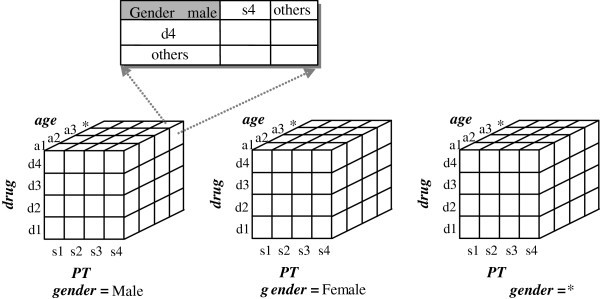
**An example a 4-D contingency cube,*C*[*Age*,*Gender*,*Drug*,*PT*].**

**Example 1** Figure 3 depicts a 4-D contingency cube, *C*[*Age*, *Gender*, *Drug*, *PT*]. The expanded cell, *C*[*Age* = *, *Gender* = Male, *Drug* = d4, *PT* = s4], illustrates the content in the form of a contingency table. In this case, *Age* can be neglected as a constraint.

Intuitively, the values of each cell can be obtained by aggregating the occurrences in the data warehouse. However, the reality is more complicated (Beyer & Ramakrishnan 1999). Firstly, the computation of the values, *b*, *c*, *d,* in the contingency table involves negative items, which are not immediately accessible. Secondly, the relationship between *Drug* and *PT* attributes is many-to-many; a simple calculation results in duplicated aggregation, making the result misleading.

The solution to the first problem is to avoid counting using negative items. Instead, the occurrence is always computed using positive items. Given a contingency table composed of the predicate, *P*_1_ = *v*_1_, *P*_2_ = *v*_2_, …, *P*_*k*_ = *v*_*k*_, *Drug* = *d* and *PT* = *s*, Table 4 details the formulae for computing all value cells, *a*, *b*, *c* and *d*, where, for simplicity, count(*v*_1_, *v*_2_, …, *v*_*k*_, *d*, *s*) denotes the occurrences of the itemset {*P*_1_ = *v*_1_, *P*_2_ = *v*_2_, …, *P*_*k*_ = *v*_*k*_, *Drug* = *d*, *PT* = *s*}. Note that each of the counts used in Table 4 indeed corresponds to a cell in the OLAP data cube, so the content of the contingency cube can be generated from the OLAP cube, without accessing the original data warehouse. Indeed, the corresponding OLAP cube supporting the aforementioned contingency table is *D*[*P*_1_, *P*_2_, …, *P*_*k*_, *Drug*, *PT*]. In this way, the OLAP cube serves two different purposes, OLAP and pattern mining, saving the cost of storing the contingency cube, without sacrificing the computational efficiency.

**Table 4:** **The formulae for the computation of each cell in a 2×2 contingency table**

Cell	The formula
*a*	count(*p*_1_, *p*_2_, … *p*_*n*_, *d*, *s*)
*b*	count(*p*_1_, *p*_2_, … *p*_*n*_, *d*) *– a*
*c*	count(*p*_1_, *p*_2_, … *p*_*n*_, *s*) *– a*
*d*	count(*p*_1_, *p*_2_, … *p*_*n*_) *– a – b – c*

#### Cube-based method for ADR detection

The proposed algorithm, namely CBM_SS (**C**ube**-B**ased **M**ining for signal detection of **S**ingle drug and **S**ingle symptom), employs a contingency cube to generate the signals of ADRs caused by drug, with/without other demographic attributes, in four phases: (1) the contingency cube extraction phase; (2) the candidate rule generation phase; (3) the measure calculation phase and (4) the signal ranking and output phase. The following describes and illustrates each of the four phases for the example shown in Table 5.

**Table 5:** **An example of a de-normalized ADR warehouse composed of six reports**

Demo_key	Year	Age	Gender	Weight	Country	Drug	PT
1	*y*_3_	*a*_2_	*g*_1_	*w*_2_	*c*_3_	*d*_1_	*s*_1_
1	*y*_3_	*a*_2_	*g*_1_	*w*_2_	*c*_3_	*d*_2_	*s*_1_
1	*y*_3_	*a*_2_	*g*_1_	*w*_2_	*c*_3_	*d*_3_	*s*_1_
2	*y*_1_	*a*_1_	*g*_2_	*w*_2_	*c*_1_	*d*_2_	*s*_2_
2	*y*_1_	*a*_1_	*g*_2_	*w*_2_	*c*_1_	*d*_3_	*s*_2_
3	*y*_2_	*a*_2_	*g*_2_	*w*_2_	*c*_2_	*d*_1_	*s*_1_
3	*y*_2_	*a*_2_	*g*_2_	*w*_2_	*c*_2_	*d*_3_	*s*_1_
3	*y*_2_	*a*_2_	*g*_2_	*w*_2_	*c*_2_	*d*_1_	*s*_2_
3	*y*_2_	*a*_2_	*g*_2_	*w*_2_	*c*_2_	*d*_3_	*s*_2_
4	*y*_1_	*a*_2_	*g*_1_	*w*_1_	*c*_3_	*d*_1_	*s*_1_
4	*y*_1_	*a*_2_	*g*_1_	*w*_1_	*c*_3_	*d*_3_	*s*_1_
5	*y*_1_	*a*_1_	*g*_2_	*w*_1_	*c*_2_	*d*_2_	*s*_1_
6	*y*_3_	*a*_2_	*g*_1_	*w*_2_	*c*_1_	*d*_2_	*s*_3_

##### Contingency cube extraction phase

According to the demographic attributes selected by users, the proposed approach selects suitable data from the stored repository of the contingency cube and loads them into the memory.

**Figure 4:**
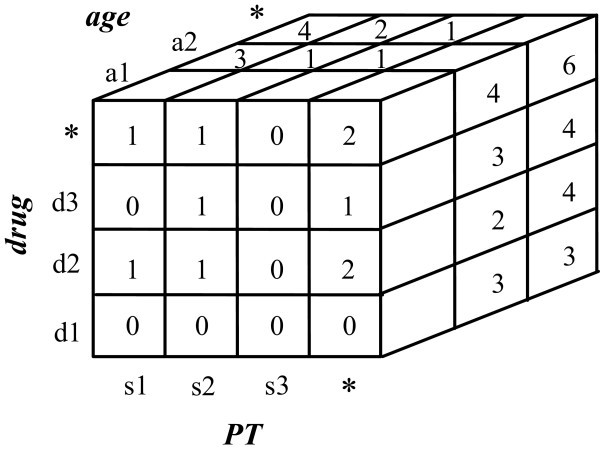
**An example a 3-D OLAP cube*D*(*Age*,*Drug*,*PT*) constructed from Table 5.**

Suppose that the content of ADR warehouse is composed of six reports, as shown in Table V, where Demo_key denotes the report ID. For convenience, all tables have been de-normalized into a single table. Further, assume that the user has specified a query, *Q*, which includes the selected demographic attribute, *Age*, and the measure, PRR, with count ≥ 3. In other words, the antecedent of the generated signals must contain age and drug information and the consequence is a symptom. In this case, the data in the OLAP cube *D*[*Age*, *Drug*, *PT*] are selected, as shown in Figure 4.

##### Rule-generation phase

**Figure 5:**
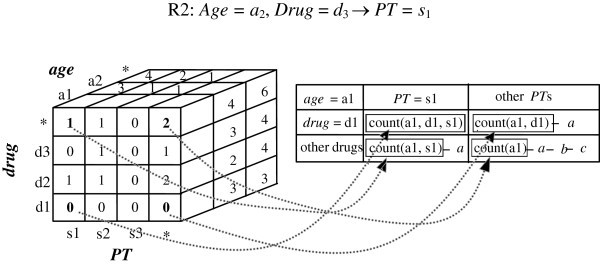
**An illustration of the correspondence between the contingency cell and the OLAP cube.**

The main purpose of this phase is to generate all of the rules from the contingency cubes. The proposed approach generates rules from each member cell in the contingency cube. That is, each cell (actually a table) in the contingency cube, with dimensions of *Drug*, *PT* and the user specified demographic, attributes generates a corresponding rule for this purpose. When the contingency cube is implemented as the OLAP cube, the corresponding OLAP cube cells required to make each contingency table are obtained, according to Table 4. In this example, the OLAP cells used to compute the contingency cell that is composed of *Age* = *a*_1_, *Drug* = *d*_1_, and *PT* = *s*_1_ are *D*[*a*_1_, *d*_1_, *s*_1_], *D*[*a*_1_, *d*_1_, *], *D*[*a*_1_, *, *s*_1_] and *D*[*a*_1_, *, *], as depicted in Figure 5. Since a threshold count ≥ 3 is specified, it is only necessary to consider those contingency cube cells with value *a* ≥ 3. The following two rules are generated:

##### Measure calculation phase

In this phase, the proposed approach examines signals whose measure exceeds the measurement threshold. For each generated rule, four values in the 2×2 contingency table, including *a*, *b*, *c* and *d*, are calculated. Then, according to the selected measurement, the measure value of each rule is calculated and then checked to determine whether it exceeds the threshold. For the rule, R1: *Age = a*_2_, *Drug* = *d*_1_ → *PT = s*_1_, the corresponding values for *a*, *b*, *c* and *d*, are calculated as follows:

Hence, PRR = 3. Similarly, the same values for R2, *a* = 3, *b* = 0, *c* = 0, *d* = 1 and PRR = 3 can be obtained.

##### Signal ranking and output phase

The proposed approach sorts all signals by their measure values and outputs all or top-*k* signals for users, in this phase. The parameter, *k,* is specified by users. In this case, both rules generated in phase 3 are output signals_._

### Associative classification based method for the detection of ADR signals

Although the cube-based method greatly reduces the computation for the detection of single drug and single symptom ADR patterns, it is awkward for use with symptoms caused by drug-drug interaction. This is because all of the dimensions of the data cube are atomic. To compute the occurrences of signals involving multiple drugs, cube cell join and compare operations must be performed; these are very time consuming. In this regard, another algorithm was developed, called ACM-MS (**A**ssociative **C**lassification based **M**ining for signal detection of **M**ultiple drugs and **S**ingle symptom).

The ACM-MS algorithm is a modified CMAR algorithm (Li et al. 2001), a recently developed associative classification algorithm. The data structure CR-tree used in CMAR is used to store compact transactions extracted from the ADR warehouse and then to generate all of the multiple-drug-single-symptom rules that satisfy the user specified measure.

**Figure 6:**
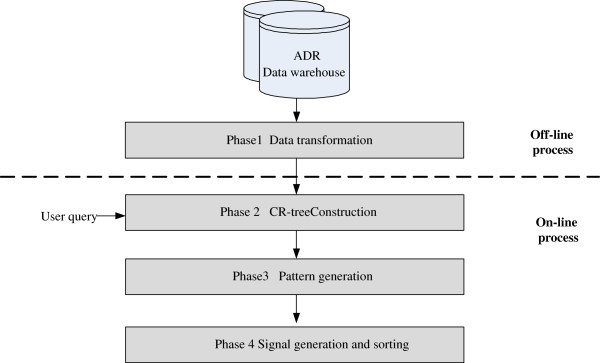
**The process workflow for the ACM-MS.**

Figure 6 shows the workflow of the ACM-MS. There are two stages: an off-line process and an on-line process, which are further divided into four phases: (1) Data transformation phase; (2) CR-tree construction phase; (3) Rule generation phase and (4) Signal generation and sorting phase. The following details the processing in each phase, with some illustrated examples.

#### Data transformation phase

In data warehouses, data is usually stored in a relational format. Most association mining algorithms, however, require that the input data take the form of a horizontal transaction type. The relational data must be transformed into a transactional data format, before mining.

This ACM-MS uses a CR-tree structure, a variant of the prefix tree that facilitates transaction compaction and in-memory computation. However, its performance is greatly affected by the ordering of the items. Indeed, previous research has shown the ordering of items by decreasing rate of occurrence yields the best performance. In this regard, each of the transformed transactions is ordered accordingly. Those infrequent items that occur less than 3 times are eliminated. The rationale is that this frequency threshold is adopted by most of the ADR signal measures and that all of the items comprising the ADR signal should be frequent and should obey the a priori principle (Agrawal & Srikant 1994).

In the example in Table 5, the set of frequent items (occurring within at least three different reports), except symptoms, is {*a*_2_: 4, *w*_2_: 4, *d*_2_: 4, *d*_3_: 4, *y*_1_: 3, *g*_1_: 3, *g*_2_: 3, *d*_1_: 3} and the set of frequent symptoms is {*s*_1_: 4}. Later it will become clear that the differentiation between symptom items and non-symptom items benefits the construction of the CR-tree. Following transaction scanning, pruning infrequent items and transaction resorting, the resulting reduced transaction dataset is shown in Table 6. Finally, the reduced transactions are stored for online processing.

**Table 6:** **The reduced transaction dataset corresponding to the example in Table**5

TID	Transaction	Class (PT) label
1	*a*_2_, *w*_2_, *d*_2_, *d*_3_, *g*_1_, *d*_1_	*s*_1_
3	*a*_2_, *w*_2_, *d*_3_, *g*_2_, *d*_1_	*s*_1_
4	*a*_2_, *d*_3_, *y*_1_, *g*_1_, *d*_1_	*s*_1_
5	*d*_2_, *y*_1_, *g*_2_	*s*_1_

#### CR-tree construction phase

**Figure 7:**
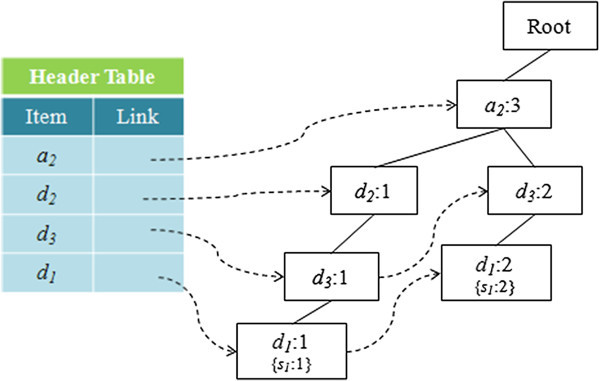
**The initial CR-tree for Table 7.**

The on-line process starts in this phase, which is activated when the user chooses to execute multidrug-ADR detection, using the system. The purpose of this phase is to construct the CR-tree from the reduced transaction dataset, using the user-specified query constraints. During the construction, any item or symptom unrelated to the user specified attributes or constraints is pruned and any transaction that does not contain any related items is eliminated. The process for construction of a CR-tree follows that used in the CMAR algorithm, but all items not in the domain of the attributes or satisfying the constraints specified in the query are pruned. If a transaction is empty or contains no related item, after item pruning, this transaction is eliminated. For example, consider the query, *Q*, used in the illustration for the algorithm, CBM-SS. Since only the attribute *age* is specified, the resulting transactions are shown in Table 7. Figure 7 shows the constructed CR-tree, wherein all symptom items are stored in the leaf nodes.

**Table 7:** **The pruned transaction dataset corresponding to the example in Table**6

TID	Transaction	Class label
1	*a*_2_, *d*_2_, *d*_3_, *d*_1_	*s*_1_
3	*a*_2_, *d*_3_, *d*_1_	*s*_1_
4	*a*_2_, *d*_3_, *d*_1_	*s*_1_

#### Pattern generation phase

In this phase, the CR-tree is traversed to determine all of the candidate rules. This process is similar to that used in the FP-growth algorithm (Han et al. 2000). That is, it constructs the conditional pattern base of each frequent item and then it builds its conditional CR-tree on the conditional pattern base. When the process for generating rules involving frequent items is complete, the item is eliminated from the header node and those nodes that indicate symptoms are merged into their parent nodes. Finally, a recursive mining procedure is performed on that conditional CR-tree, in order to generate all patterns, except those that do not contain any related items.

**Figure 8:**
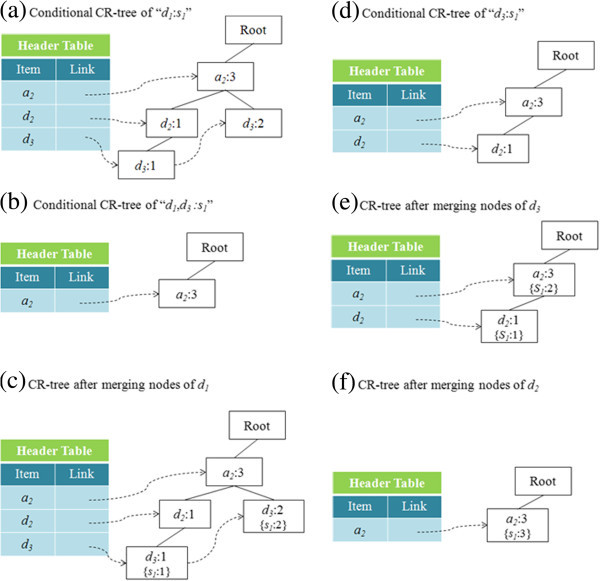
**All of the conditional CR-trees generated from Figure 6.**

Consider the example in Figure 7. The process for frequent pattern generation is shown in Figure 8. The frequent patterns are shown in Table 8. All of the frequent patterns are checked if they contain items that satisfy the user-specified attributes, which in this example is *Age*. The resulting set of satisfactory patterns is shown in Table 9.

**Table 8:** **The frequent patterns generated from the CR-tree in Figure**7

Frequent patterns	Count
*Drug* = *d*_1,_*Symptom = s*_1_	3
*Drug*_*1*_ = *d*_1_, *Drug*_2_ = *d*_*3*,_*Symptom = s*_1_	3
*Age = a*_2_, *Drug* = *d*_1,_*Symptom = s*_1_	3
*Age = a*_2_, *Drug*_1_ = *d*_1_, *Drug*_2_ = *d*_3,_*Symptom = s*_1_	3
*Drug* = *d*_3,_*Symptom = s*_1_	3
*Age = a*_2_, *Drug* = *d*_3_, *Symptom = s*_1_	3
*Age = a*_2_, *Symptom = s*_1_	3

**Table 9:** **The resulting frequent patterns from Table**8**that contain the attribute,*Age***

Frequent patterns	Count
*Age = a*_2_, *Drug* = *d*_1,_*Symptom = s*_1_	3
*Age = a*_2_, *Drug*_1_ = *d*_1_, *Drug*_2_ = *d*_3,_*Symptom = s*_1_	3
*Age = a*_2_, *Drug* = *d*_3_, *Symptom = s*_1_	3
*Age = a*_2_, *Symptom = s*_1_	3

#### Signal generation and sorting phase

The purpose of this phase is to generate signals of interest. Each pattern generated in the previous phase that satisfies the user-specified conditions corresponds to a candidate rule with a symptom in the consequence, with the other items being the antecedent. The four cell values, *a*, *b*, *c* and *d*, required to calculate the rule’s measure, are immediately available from the counts of other sub-patterns of the candidate rule. The algorithm then determines all of the rules whose measures exceed or are equal to the threshold. These signals are sorted in accordance with their measures and the number of items in the antecedent. Finally, all qualified or the top-*k* signals are output. If the user is not satisfied with the results, the query can be re-specified, which sets the ACM-MS back to phase 3, to execute the modified query analysis.

In this example, only one mutidrug-ADR signal is output, as shown in Table 10, where SE represents standard error.

**Table 10:** **The measured values of the signals**

Rule	***a***	***b***	***c***	***d***	PRR	PRR −1.96SE>1
*Age = a*_2_, *Drug*_1_ = *d*_1_, *Drug*_2_ = *d*_3__→_*Symptom = s*_1_	3	0	0	1	3	Yes

### System functionalities and experiments

All of the experiments were performed on a personal computer with an Intel Core2 Duo 2.33Ghz CPU, 3GB main memory and a 320 GB hard disk, running Windows XP. The DBMS used was Microsoft SQL SERVER 2005. All system interfaces were implemented using Microsoft ASP.NET 2.0.

#### System interface and functionalities

**Figure 9:**
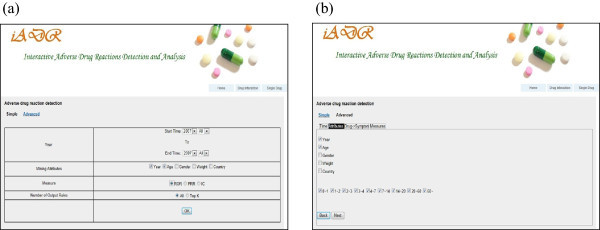
**System snapshots of two modes of the query interface - simple mode and advanced mode.**

The system provides two types of ADR signal detection; drug-ADR and multidrug-ADR, via the two menus, Single Drug and Drug Interaction, respectively. Each menu further supports two modes of query interface; simple and advanced. As shown in Figure 9(a), the simple mode analysis interface provides general signal detection (neither specific drug nor symptom can be designated) from target data, with the dimensions of the years of the data reported (in continuous range) and any combinations of demographic attributes (Age, Gender, Weight and Country). As indicated in the survey conducted by Deshpande et al. ( 2010), there is considerable variation in the definition of a significant alert for ADR signals for different signal detection methods and no specific metric is better than all of the others; each has its strengths and limitations (Bate & Evans 2009; Roux et al. 2005). As such, it is necessary to consider more than one metric/threshold combination within a given study. The proposed system supports three measures commonly used in the pharmacovigilance community, i.e., ROR, PRR and IC, and two choices of output display; all signals or only the top *k* signals.

**Figure 10:**
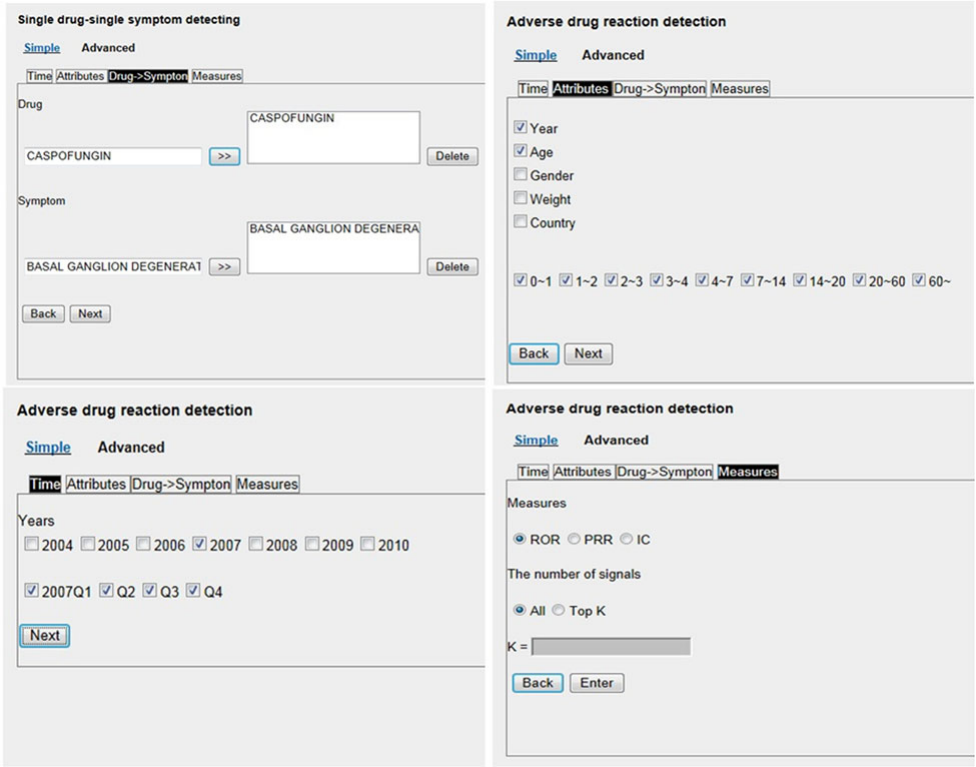
**An illustration of an advanced query specification, in terms of drug/symptom, demographic attributes, time and measure.**

However, the advanced interface mode (Figure 9(b)) allows the detection of ADR signals associated with a specific drug and/or symptom, with the option of a specified constraint using any combination of the demographic attributes and the time of the reported data. Figure 10 shows an example query specification, in terms of drug/symptom, time, demographic attributes and measure, respectively. Note that in this mode, the history of the target data can be specified for several discontinuous time intervals.

**Figure 11:**
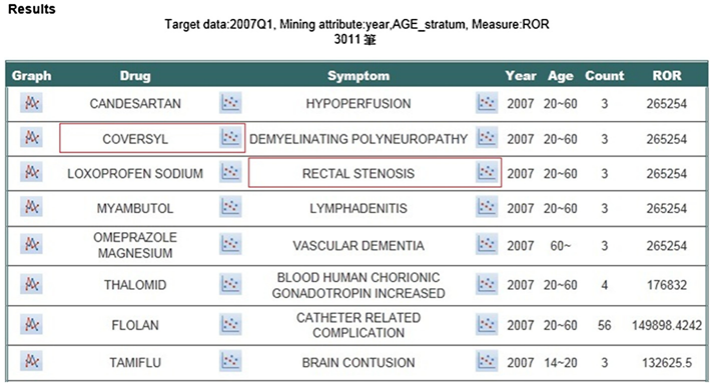
**A snapshot of the signal output display.**

**Figure 12:**
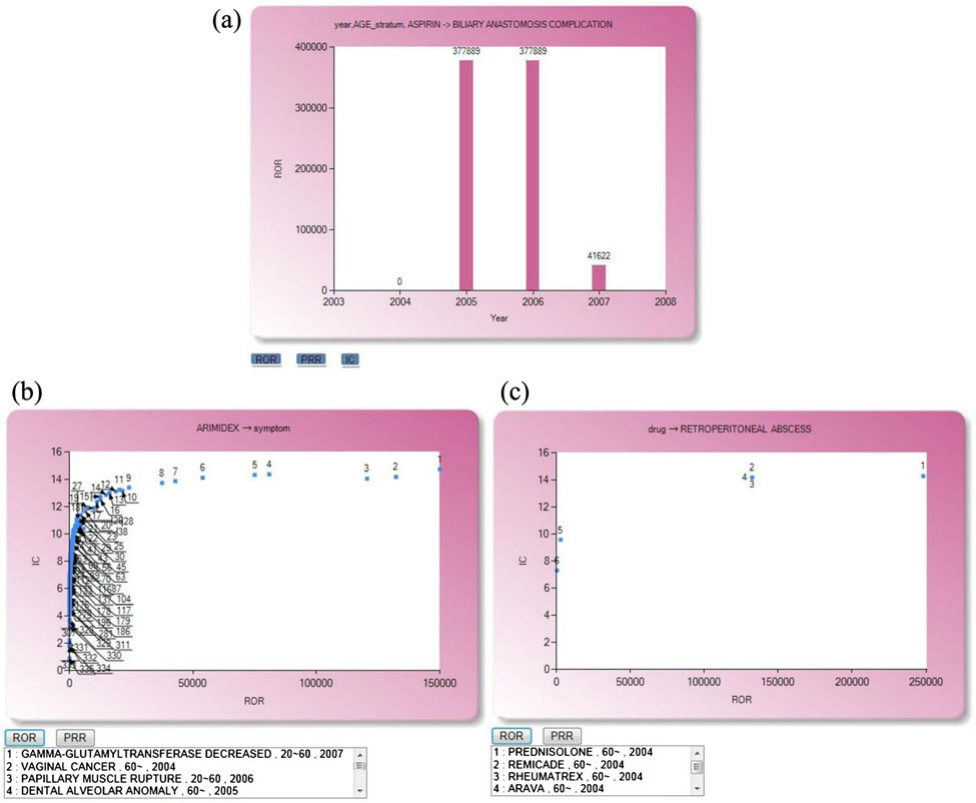
**Snapshots of the visualization functions supported by iADR: (a) an analysis of signal change over time; (b) a comparison of symptoms associated with a specific drug and (c) a comparison of drugs associated with a specific symptom.**

In Figure 11, the output signals after query execution are listed in the form of a rule table, for drug, symptom, time, the demographic attributes specified in the query, count, and the user’s preferred measure. There are three clickable visualization icons for each rule, one in the front of the rule and the other two beside the corresponding drug and symptom, respectively. The first icon allows visualization of the trend analysis of the corresponding ADR signal over the observed duration, displayed in either one of the three measures. The icon beside the drug allows the user to inspect and compare the associated symptoms, with the y-axis fixed to IC while the x-axis can represent either ROR or PRR. The last icon beside the symptom allows a similar visualization with the focus on the associated drugs. Figure 12 shows a snapshot of these three visualization displays.

#### Experiments

##### Performance study

Experiments were conducted to study the performance of the two proposed algorithms, CBM-SS and ACM-MS, for various sizes of data set. The efficiency was evaluated using the data reported by the FDA in 2007, which concerns 14,437 drugs, 10,436 symptoms and 230 demographic values. This dataset was further decomposed into five subsets, each containing a different number of transactions, namely T10K, T50K, T100K, T150K and T200K.

**Figure 13:**
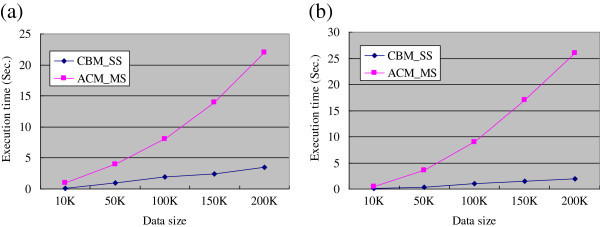
**Performance evaluation of CBM_SS and ACM_MS on the 2007 AERS dataset: (a) with no demographic attributes; (b) with all demographic attributes.**

No specific drug and symptom were designated for this experiment, in order to see how the algorithms perform in response to such a general query. Two query conditions were considered: No demographic attributes are selected and the other extreme case, for all demographic attributes, is considered in the query. As the results in Figure 13 demonstrate, both algorithms perform satisfactorily, even for this rigid query setting, and show almost linear scalability. CBM_SS is significantly faster than ACM_MS, consuming only a few seconds in all cases.

##### Experimental results for signal detection

The effectiveness of the proposed algorithms was studied by comparing the resulting ADR signals with those reported in medical documents (DrugDigest 2012; PubMed 2012). The last quarter of 2007 AERS dataset was used for these experiments.

Firstly, the drug-ADR signals generated by CBM-SS are considered. Two different drugs, CAPTOPRIL and RANITIDINE, were examined to determine the discovery results. For the drug, CAPTOPRIL, no demographic attribute is considered, while for RANITIDINE all demographic attributes are included.

Two hundred and sixty-three signals contain distinct symptoms related to CAPTOPRIL. Table 11 lists the top 10 signals, ranked by their ROR measure. According to the description in Drug Digest (2012), “*CAPTOPRIL is an ACE inhibitor. This medicine is used to treat high blood pressure and heart failure. It is used to treat heart damage after a heart attack. It can also slow the progression of kidney disease in diabetic patients*.” Another document reported that the symptoms, BASAL GANGLION DEGENERATION and LARGE INTESTINAL OBSTRUCTION, can be treated by CAPTOPRIL in association with other drugs. Thus, it is very likely that BASAL GANGLION DEGENERATION (No. 1), LARGE INTESTINAL OBSTRUCTION (No. 3) and HYPERTROPHIC CARDIOMYOPATHY (No. 7) are noises. In addition, no relevant documents report INJECTION SITE PHLEBITIS (No. 4) and JAW FRACTURE (No. 8) as being related to CAPTOPRIL. These two signals require further professional analysis and literature validation. The other remaining signals are the adverse drug reactions of CAPTOPRIL.

**Table 11:** **TOP-10 suspected ADR signals associated with CAPTOPRIL**

No.	Symptom	ROR value	Count
1	BASAL GANGLION DEGENERATION	1336.8434	6
2	OESOPHAGEAL INFECTION	398.9214	3
3	LARGE INTESTINAL OBSTRUCTION	222.4044	5
4	INJECTION SITE PHLEBITIS	181.325	3
5	NODAL ARRHYTHMIA	97.8079	6
6	VIRAL UPPER RESPIRATORY TRACT INFECTION	79.78	3
7	HYPERTROPHIC CARDIOMYOPATHY	78.3525	4
8	JAW FRACTURE	62.3452	12
9	PALMAR ERYTHEMA	62.327	3
10	PANCREATIC NEOPLASM	62.327	3

Seventy-eight signals are related to RANITIDINE. Table 12 lists the top 10 signals, ranked by their IC value. A document from the Drug Digest reported that, “*RANITIDINE is a type of antihistamine that blocks the release of stomach acid. It is used to treat stomach or intestinal ulcers. It can relieve ulcer pain and discomfort, and the heartburn from acid reflux*.” From this description, it can be seen that RANITIDINE is often used in the treatment of stomach or intestinal ulcers. Of the top 10 suspected signals, it is seen that NEUTROPENIC COLITIS (No. 2) resembles a noise, since it is related to intestinal ulcers. The other nine signals are recorded in the literature as the adverse drug reactions for RANITIDINE. In addition, MENINGITIS BACTERIAL is an ADR that is easily caused in young people by RANITIDINE.

**Table 12:** **TOP-10 suspected ADR signals with demographic attributes associated with RANITIDINE**

No.	Age	Gender	Weight	Symptom	IC value	Count
1	14~20	M	54.0~	FLUID IMBALANCE	13.2358	3
2	4~7	F	10.0~15.0	NEUTROPENIC COLITIS	12.0983	5
3	7~14	M	30.0~40.0	MALIGNANT HYPERTENSION	11.3988	3
4	~1	F	~2.5	CATHETER SEPSIS	11.1289	3
5	14~20	M	54.0~	MENINGITIS BACTERIAL	11.0783	3
6	20~60	M	54.0~	PO2 DECREASED	10.5905	6
7	7~14	M	30.0~40.0	CAPILLARY EAK SYNDROME	10.497	3
8	60~	F	54.0~	LARYNGEAL OEDEMA	9.8955	3
9	7~14	M	30.0~40.0	REVERSIBLE POSTERIOR LEUKOENCEPHALOPATHY SYNDROME	9.1076	3
10	20~60	F	54.0~	PUPIL FIXED	9.095	3

Next the multidrug-ADR signals generated by ACM-MS are considered. The drug pair, AREDIA and ZOMETA, was used for this examination. Nine ADR signals are generated, and these are ranked by their PRR value, as shown in Table 13. No relevant documents report TROPONIN I INCREASED (No. 2) and ILEUS (No. 4) as being related to AREDIA and ZOMETA. These two signals require further professional analysis and literature validation. Other ADRs are either the symptoms of treatment or the adverse drug reactions reported in the literature.

**Table 13:** **Suspected ADR signals associated with the drug set, {AREDIA, ZOMETA}**

No.	Symptom	PRR value	Count
1	WOUND TREATMENT	2711.547	22
2	TROPONIN I INCREASED	1182.094	22
3	TOOTHACHE	93.3415	30
4	ILEUS	66.2488	26
5	WHEEZING	38.2239	30
6	LUNG DISORDER	38.0937	32
7	VISUAL DISTURBANCE	19.4125	34
8	RESPIRATORY FAILURE	10.4387	22
9	HYPOTENSION	4.8558	20

## Conclusions

This paper develops a system platform for the analysis and detection of adverse drug reactions. Users can interact with this platform to examine various forms of ADR signals from different viewpoints, by selecting and re-adjusting parameters and measures of interest. Two efficient algorithms, CBM-SS and ACM-MS, were respectively used to facilitate the discovery of drug-ADR and multidrug-ADR patterns. A preliminary experiment using selected AERS data demonstrates the efficacy and efficiency of the proposed algorithms.

One of the main problems encountered in pharmacovigilance using computer systems is a lack of standard measures for signal detection. Signal detection extracts noteworthy, suspicious ADRs in advance. However, filtering of the noises relies on the thresholds and determination of suitable thresholds for the different methods that allows the discovery of valuable information is not a robust procedure. Thus, data mining methods cannot replace traditional methods of signal detection, but only serve as an auxiliary tool. Assessment of the reliability of the detected signals requires further professional analysis and literature validation.

This paper presents a preliminary development of ADR detection and analysis and there is much scope for future research, such as:

1. The provision of other forms of ADR signals: Currently, this iADRs system only discovers drug-ADR and multidrug-ADR signals. The mining algorithms will be improved to accommodate other forms of signals, including drug-multiADR and multidrug-multiADR signals, and even to consider other information, such as drug dose and onset latency, in order distinguish between types of ADR, i.e., those caused by drug toxicity or by a patient’s hypersenstivity (drug allergy) (Wongpoowarak & Wongpoowarak 2002).
2. The inclusion of more functions: It is planned that more visualization tools will be added, as well as signal tracking and monitoring mechanisms, so that users can track the change in some specific ADR signal, as reports accumulate over time, or can set an automatic alert when a monitored drug generates noticeable ADR signals.
3. Integration with drug target ontology: Most drugs act by binding to a specific protein, namely the drug target, in order to change its biochemical or biophysical activities (Lin et al. 2009). The incorporation of drug target knowledge with the detection of adverse drug reactions may provide clues for an explanation. The next step is the integration of drug target ontology into this system, in order to allow an in-depth investigation of adverse drug reactions.
